# Beta-Rhythm Oscillations and Synchronization Transition in Network Models of Izhikevich Neurons: Effect of Topology and Synaptic Type

**DOI:** 10.3389/fncom.2018.00059

**Published:** 2018-08-14

**Authors:** Mahsa Khoshkhou, Afshin Montakhab

**Affiliations:** Department of Physics, College of Sciences, Shiraz University, Shiraz, Iran

**Keywords:** beta oscillations, synchronization, Izhikevich neuron, synapse, neural network, complex networks, phase transition

## Abstract

Despite their significant functional roles, beta-band oscillations are least understood. Synchronization in neuronal networks have attracted much attention in recent years with the main focus on transition type. Whether one obtains explosive transition or a continuous transition is an important feature of the neuronal network which can depend on network structure as well as synaptic types. In this study we consider the effect of synaptic interaction (electrical and chemical) as well as structural connectivity on synchronization transition in network models of Izhikevich neurons which spike regularly with beta rhythms. We find a wide range of behavior including continuous transition, explosive transition, as well as lack of global order. The stronger electrical synapses are more conducive to synchronization and can even lead to explosive synchronization. The key network element which determines the order of transition is found to be the clustering coefficient and not the small world effect, or the existence of hubs in a network. These results are in contrast to previous results which use phase oscillator models such as the Kuramoto model. Furthermore, we show that the patterns of synchronization changes when one goes to the gamma band. We attribute such a change to the change in the refractory period of Izhikevich neurons which changes significantly with frequency.

## 1. Introduction

Synchronization is an important collective phenomenon that may emerge in locally interacting physical and biological oscillatory systems (Pikovsky et al., 2001; Barahona and Pecora, 2002; Motter et al., 2005; Arenas et al., 2008). Neural tissue of central nervous system can generate oscillatory activity in various scales from individual neuron firing to macroscopic oscillations in large neural ensembles (Engel et al., 2001; Varela et al., 2001; Buzsaki and Draguhn, 2004; Jensen and Lisman, 2005; Buzsaki, 2006). Macroscopic rhythmic activity which is observed in electroencephalography (EEG) recordings, is believed to occur due to emergence of synchronization in oscillations of constituent neurons. Synchronization of neural activity has a fundamental role in brain functions such as vision, memory, action, perception, information transfer, thought, and so on (Kandel et al., 2000; Steinmetz et al., 2000; Fell and Axmacher, 2001; Fries et al., 2001; Varela et al., 2001; Uhlhass et al., 2008; Jiang et al., 2012).

Neural oscillations have been documented to cover a broad spectrum of frequencies. These oscillations are observed widely in every level of central nervous system and are usually categorized into five frequency bands: delta 0.5−3.5 Hz, theta 4−7 Hz, alpha 8−12 Hz, beta 13−30 Hz, and gamma >30 Hz (Buzsaki, 2006; Rosanova et al., 2009). Beta rhythms are associated with normal wakeful consciousness states and appear when one is alert, attentive or when a person is engaged in problem solving or decision making. Beta waves are also associated with the activities of motor cortex (Baker, 2007; Engel and Fries, 2010).

Synchronization in neural population has been in the focus of intense experimental and theoretical research recently. See (Salinas and Sejnowski, 2001; Bennett and Zukin, 2004; Zhou et al., 2006; Mikkelsen et al., 2013; Sadeghi and Valizadeh, 2014; Matias et al., 2015; Esfahani et al., 2016; Pazó and Monthrió, 2016) for a few examples. Although beta-band activities have a significant role in brain functions, they have attracted less attention than other frequency bands (Engel and Fries, 2010). This is all the more important as many fundamental functions of the brain are associated with such oscillations. For example, synchronization transition is an important issue. From a theoretical point of view, synchronization in a neuronal network occurs as one increases synaptic strength. How this transition occurs is of fundamental importance. Generally, the transition can occur either as a continuous transition or a discontinuous (explosive) manner. If continuous, a small change can lead to small changes in systems response; however, if explosive, a small change can lead to dramatic changes in system's response. In addition to the type of synaptic interaction, the role of network topology is of key issue in determining the order of synchronization transition. In this paper, we intend to investigate the effect of network topology and synaptic type on synchronization phase transition in populations of spiking neurons with none-identical intrinsic frequencies in beta band. Specifically, we will focus on the order of the emerging phase transition for various network structures and different synaptic interactions. It is believed that normal brain activity requires it to be close to a phase boundary (a critical point) which consequently provide access to both synchronous and asynchronous oscillations with small change in the input (Beggs and Timme, 2012; Hesse and Gross, 2014; Moosavi et al., 2017). Hence, it is important to know whether the emerging synchronization transition is continuous or abrupt.

It is usual to evoke phase oscillators to characterize transition properties of neural oscillations. See (Shimokawa and Shinomoto, 2006; Cumin and Unsworth, 2007; Maistrenko et al., 2007; Kitzbichler et al., 2009; Gómez-Gardeñes et al., 2010; Botcharova et al., 2014; Timms and English, 2014) for some examples. While this choice offers many computational and analytic advantages, it suffers from some drawbacks. For example, it is not possible to consider a biologically realistic dynamical model as a phase oscillator, since many important features such as realistic synaptic interaction, are not easily implemented in phase oscillator models such as the Kuramoto model. Also, the spiking patterns of real neurons with wide range of frequencies are washed out in phase oscillator models. We therefore propose to study neuronal dynamics according to the Izhikevich model (Izhikevich, 2003) which is obtained by reducing some biological aspects of Hodgkin-Huxley (HH) neuron using bifurcation methods (Izhikevich, 2007). This model is computationally simpler than HH neuron, but is still biologically plausible.

To describe the functional form of synaptic interactions, we use two experimentally documented synaptic types: electrical synapses or gap junctions and chemical synapses (Kopell and Ermentrout, 2004; Simon et al., 2005; Sohal and Huguenard, 2005; Roth and van Rossum, 2009; Pérez et al., 2011; Kuo et al., 2016). These two types of interactions will appear as distinct expressions for synaptic currents to be added to the Izhikevich neurons. To describe the structure of synaptic interaction, we couple neurons via a network. It is well-known that network connectivity can have strong effects on patterns of collective behavior such as synchronization (Watts and Strogatz, 1998; Strogatz, 2001). It is believed that key elements such as small-world effect, clustering, and heterogeneity are of fundamental importance effecting the general collective behavior of a network. We therefore propose to study various network structures starting with a regular ring with high clustering and no randomness. We next consider small-world networks which provide a balance between high clustering and small-world effect. We also consider the more random structures such as Erdos-Renyi (homogeneous) and scale-free (heterogeneous) networks with low clustering but dense long-range synapses.

Our main results are as follows: (i) we find that electrical synapses are more conducive to synchronization than chemical synapses, leading to explosive synchronization in beta band in random networks. (ii) we find that the effect of clustering is far more important than small-world effect in determining the order of transition. (iii) we find that patterns of synchronization are distinctly different in beta band from the corresponding transitions in the high frequency gamma band.

## 2. Methods

To construct a neural circuit, we consider *N* Izhikevich neurons on an arbitrary network with a specific (symmetric) adjacency matrix *A*. The electrical activity of each neuron of this ensemble is described by a set of two ordinary nonlinear coupled differential equations (Izhikevich, 2003):

(1) $$\begin{array}{l} \frac{dv_{i}}{dt}=0.04v_{i}^{2}+5v_{i}+140-u_{i}+I_{i}^{DC}+I_{i}^{syn} \end{array}$$

(2) $$\begin{array}{l} \frac{du_{i}}{dt}=a\left(bv_{i}-u_{i}\right) \end{array}$$

with the auxiliary after-spike reset:

(3) $$\begin{array}{l} if\;v_{i}\geq 30,\;then\;v_{i}\;\to \;c\;and\;u_{i}\;\to \;u_{i}+d \end{array}$$

for *i* = 1, 2, …, *N*. Here *v*_*i*_ is the membrane potential and *u*_*i*_ is the membrane recovery variable. When *v*_*i*_ reaches its apex (*v*_*max*_ = 30 mV), voltage and recovery variable are reset according to Equation (3). The term $\left(0.04v_{i}^{2}+5v_{i}+140\right)$ has been chosen so that *v* has mV unit and *t* has ms units (Izhikevich, 2003). In addition *a*, *b*, *c*, and *d* are four adjustable parameters in this model. Tuning these parameters, Izhikevich neuron is capable of reproducing about twenty different intrinsic firing patterns observed in real neurons (Izhikevich, 2006; Izhikevich and Edelman, 2008). In this paper we set *a* = 0.02, *b* = 0.2, *c* = −65, and *d* = 8, which corresponds to regular spiking pattern (Izhikevich, 2003, 2006).

The term $I_{i}^{DC}$ is an external current which determines intrinsic firing rate of uncoupled Izhikevich neurons. Regularly spiking Izhikevich neurons exhibits a Hopf bifurcation at *I*^*DC*^ = 3.78 (Kim and Lim, 2013). We choose values of $I_{i}^{DC}$ randomly from a Poisson distribution with mean value 10. Thus the intrinsic firing rates *f*_*i*_ lay in beta band and are different from one neuron to the other. The term $I_{i}^{syn}$ in Equation (1) denotes synaptic current received by post-synaptic neuron *i*. If the synapse is electrical, the synaptic current is (Roth and van Rossum, 2009; Pérez et al., 2011):

(4) $$\begin{array}{l} I_{i}^{syn}=\frac{1}{D_{i}}\underset{j}{\sum }g_{ji}\left(v_{j}-v_{i}\right) \end{array}$$

and if the synapse is chemical then (Roth and van Rossum, 2009; Pérez et al., 2011):

(5) $$\begin{array}{l} I_{i}^{syn}=\frac{1}{D_{i}}\underset{j}{\sum }g_{ji}\frac{exp\left(-\frac{t-t_{j}}{\tau_{s}}\right)-exp\left(-\frac{t-t_{j}}{\tau_{f}}\right)}{\tau_{s}-\tau_{f}}\left(V_{0}-v_{i}\right) \end{array}$$

where *D*_*i*_ is in-degree of node *i*, *g*_*ji*_ is the strength of synapse from pre-synaptic neuron *j* to post-synaptic neuron *i. g*_*ji*_ = *gA*_*ji*_, where *g* is the electrical conductance of synapse and *A*_*ji*_ is the element of adjacency matrix of the underlying network (Gros, 2015). *A*_*ji*_ = 1 if nodes *j* and *i* are connected and *A*_*ji*_ = 0, otherwise. Also in Equation (5) τ_*s*_ = 1.7 and τ_*f*_ = 0.2 are the slow and fast synaptic decay constants (Roth and van Rossum, 2009), *t*_*j*_ is the instance of last spike of pre-synaptic neuron *j* and *V*_0_ is the reversal potential of synapse which is equal to zero since we assumed that all synapses in our circuit are excitatory. We only consider networks which are composed of one giant cluster, and thus no isolated nodes or clusters exist.

Our main goal is to study the role of various network properties on synchronization patterns that may emerge. The leading network properties we consider are clustering coefficient (*C*) and average path length (*L*). Also, the existence of hubs in heterogeneous networks are thought to play an important role in synchronization. We consider a regular ring with high clustering and large average path length (large-world-effect), a slightly random network which preserves clustering but has small-world effect, as well as two random networks which exhibit small clustering but strong small-world effect: a homogeneous Poissonian network with no hub as well as a heterogeneous scale-free network with hubs. Details of the networks used and comparison with corresponding theoretical values are summarized in Table 1.

**Table 1:** Theoretical values of clustering coefficient *C* and its corresponding measured values for the four network structures we have used in this study, first and second columns.

**Network**	***C*_*t*_**	***C*_*m*_**	***L*_*t*_**	***L*_*m*_**
Ring	0.734	0.734	10.5	10.5
WS	–	0.730	–	3.83
ER	0.048	0.054	1.77	2.06
SF	0.020	0.026	2.85	2.60

*The size of all networks is N = 1, 000 and the coordination number is z ≃ 50 except for SF network where z ≃ 20. Similar results for average path length L are also shown in the third and fourth columns. Note that the theoretical values for WS network depend on the density of long-range links p and is not available in the given closed form, see Figure 3A for more details. Theoretical calculations are performed by using formulas in (Gros, 2015)*.

We integrate the dynamical equations using fourth-order Runge-Kutta method with a time step of 0.01 ms in order to obtain *v*_*i*_(*t*). We typically evolve the entire system for a long time and make sure that the system has reached a stationary state. We then perform our measurements and calculations. We obtain the instants of firings of all neurons and then assign an instantaneous phase to each neuron between each pairs of successive spikes, as in Pikovsky et al. (1997):

(6) $$\begin{array}{l} \phi_{i}\left(t\right)=2\pi \frac{t-t_{i}^{m}}{t_{i}^{m+1}-t_{i}^{m}} \end{array}$$

where $t_{i}^{m}$ is the instant of *m*^*th*^ spike of neuron *i*. We define a global instantaneous order parameter:

(7) $$\begin{array}{l} S(t)=\frac{2}{N(N-1)}\sum_{i\neq j}cos^{2}\left(\frac{\phi_{i}(t)-\phi_{j}(t)}{2}\right) \end{array}$$

where the sum is over all pairs of neurons in the system whether they are connected or not. The global order parameter *S* is the long-time-average of *S*(*t*) in the stationary state of the system (*S* = 〈*S*(*t*)〉_*t*_) and measures the collective phase synchronization in neuronal oscillations. *S* is bounded between 0.5 and 1. If neurons spike out-of-phase, then *S* ≃ 0.5, where they spike completely in-phase *S* ≃ 1 and for states with partial synchrony 0.5 < *S* < 1. Synchronization transition is displayed in *S*−*g* plots where transition is expected to occur at a given value of *g*. We note that we have also calculated the Kuramoto order parameter and have found identical results as the ones calculated using Equation (7). The relevant codes have been shared in public domain at figshare.com.

## 3. Results

**Figure 1:**
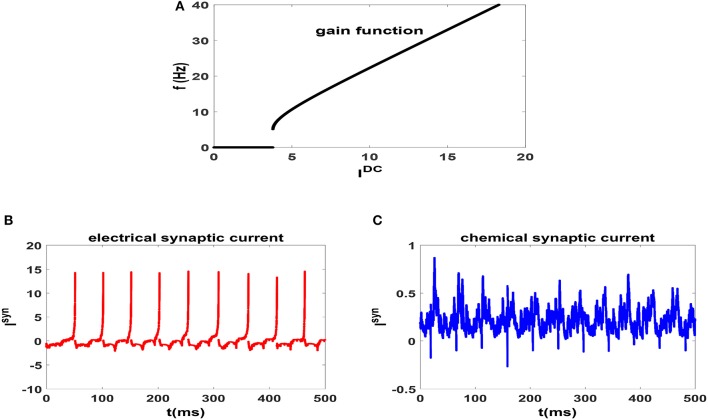
**(A)** Gain function of Izhikevich neuron which shows the dependence of firing rate of an uncoupled neuron to the external current. **(B)** Electrical [and **(C)** Chemical] synaptic current which an exemplary neuron in a network receives for *g* = 0.15. In this case the neurons of the network are unsynchronized. *t* = 0 indicates the beginning of stationary state.

In Figure 1A, we have plotted the gain function of regularly spiking Izhikevich neuron. Gain function of a neuron shows the dependence of firing rate on the external stimulating current (Gerstner et al., 2014). It is seen that the neuron shows type II excitability in this parameter regime and is capable of generating regular spikes with a broad range of intrinsic frequencies from theta-band to gamma-band. This range is more diverse than the possible range of firing rates of HH neuron (Lee et al., 1998). Also for an illustration, we have plotted the time dependence of the electrical and chemical synaptic currents which an exemplary neuron in a network receives from its neighbors at the beginning of stationary state in Figures 1B,C, respectively. Here *g* = 0.15 and neurons of the circuit are unsynchronized. We note that the pattern of electrical synaptic current is very different from that of the chemical synaptic current. For one thing, it is an order of magnitude stronger (15 vs. 0.5). Secondly, they are dispersed and act more as a pulse as opposed to fluctuating current due to chemical synapses. Thus it is expected that electrical synapses have more impact on emergence of synchronization in the system.

**Figure 2:**
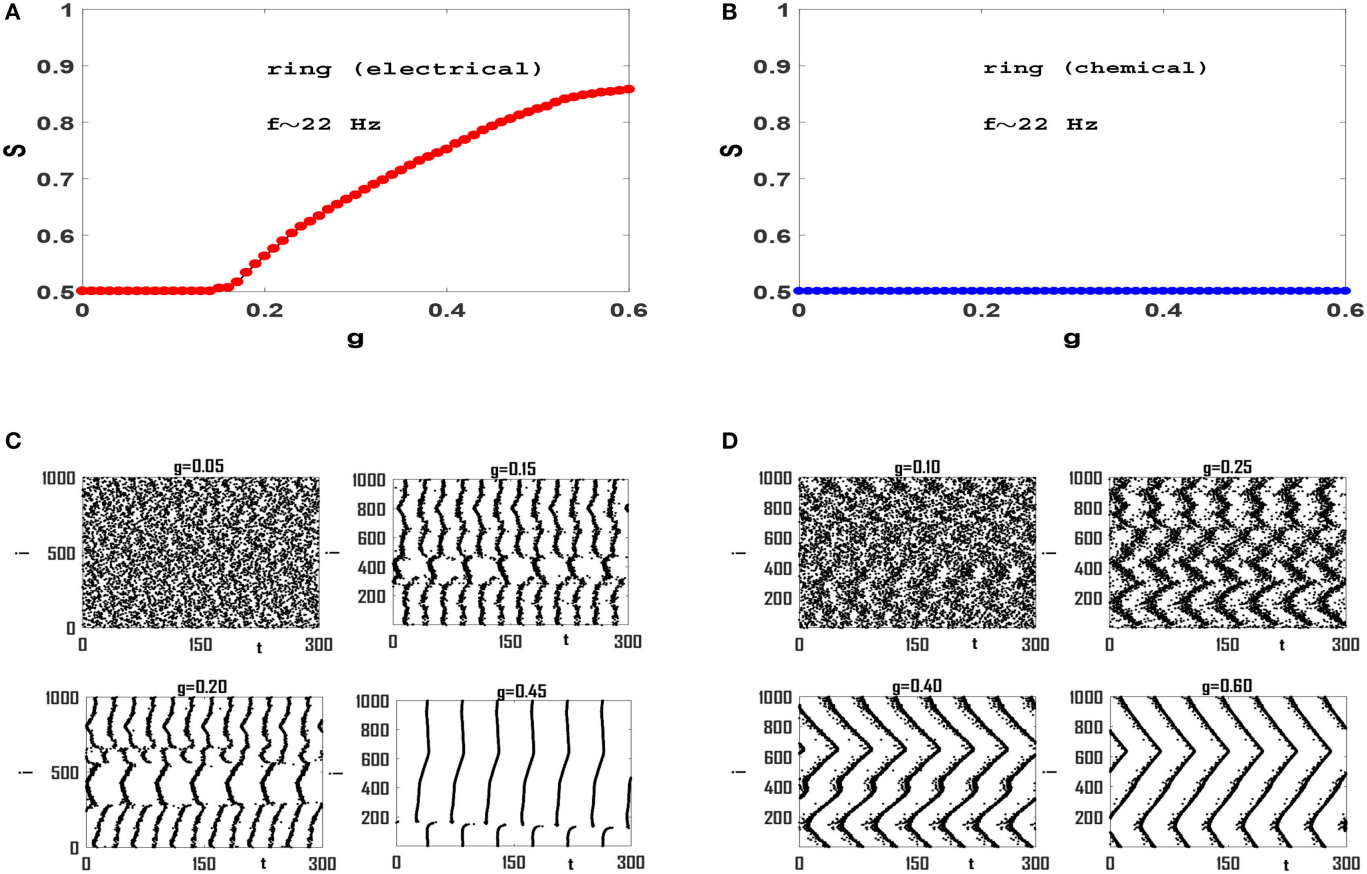
Synchronization diagram of Izhikevich neurons on a regular ring for: **(A)** circuit with electrical synapses and **(B)** circuit with chemical synapses. **(C)** Raster plots of the system of panel **(A)** for four different values of *g*. **(D)** Raster plots of the system in **(B)** for four different values of *g*. The mean firing rate is *f* ≃ 22 Hz here, and *t* = 0 indicates the beginning of stationary state. *t* is measured in units of *ms*.

Next we focus on synchronization transition in network models. We construct networks of size *N* = 1, 000 and coordination number (average connectivity) *z* = 50, unless otherwise stated. Also we set the values of $I_{i}^{DC}$ so that the intrinsic firing rates *f*_*i*_ are in beta-band and have mean-value *f* = 〈*f*_*i*_〉 ≃ 22 Hz, unless otherwise stated. Synchronization diagrams for regular ring of Izhikevich neurons with electrical and chemical synaptic interactions are illustrated in Figures 2A,B, respectively. It is observed that the network with electrical synapses exhibits a continuous transition to phase synchronization, while no transition occurs in the network with chemical synapses. Investigation of raster plots of the system with electrical synapses (Figure 2C) reveals that when synaptic interaction is weak, neurons spike out-of-phase. Note that the mean firing rate is *f* ≃ 22 Hz, and therefore each neuron should fire about seven times in the 300 ms window that is illustrated here. Increasing *g* slightly, leads to two neural groups each of which contains neurons that spike partially coherently but the members of two groups spike anti-phase with respect to each other. See *g* = 0.15 in Figure 2C. When we increase *g*, the phases of a number of members in one group gradually match the phases of the members of the other group. Hence the order parameter of synchronization increases continuously from *S* = 0.5 to higher values and the neural network exhibits a continuous transition to phase synchronization. In case of ring with chemical synapses, no anti-phase groups form. Since neuronal interactions are local, increasing synaptic strength, leads to the formation of wave-like pattern in order of neuronal spikes (Figure 2D). Although increasing *g* enhances local coherence in neuronal oscillations and each neuron has a small phase lag with adjacent neurons in the circuit, there exists no global order in the network and *S* ≃ 0.5, see Figure 2D. We cannot increase *g* to arbitrary large values, as after a certain value (near *g* ≃ 0.6) neurons start bursting instead of regular spiking (Kim and Lim, 2013). We therefore conclude that spiking Izhikevich neurons with chemical synapses with purely local interactions lead to local order without any long-range order necessary for a phase transition. However, the effect of long-range interaction may change this picture.

**Figure 3:**
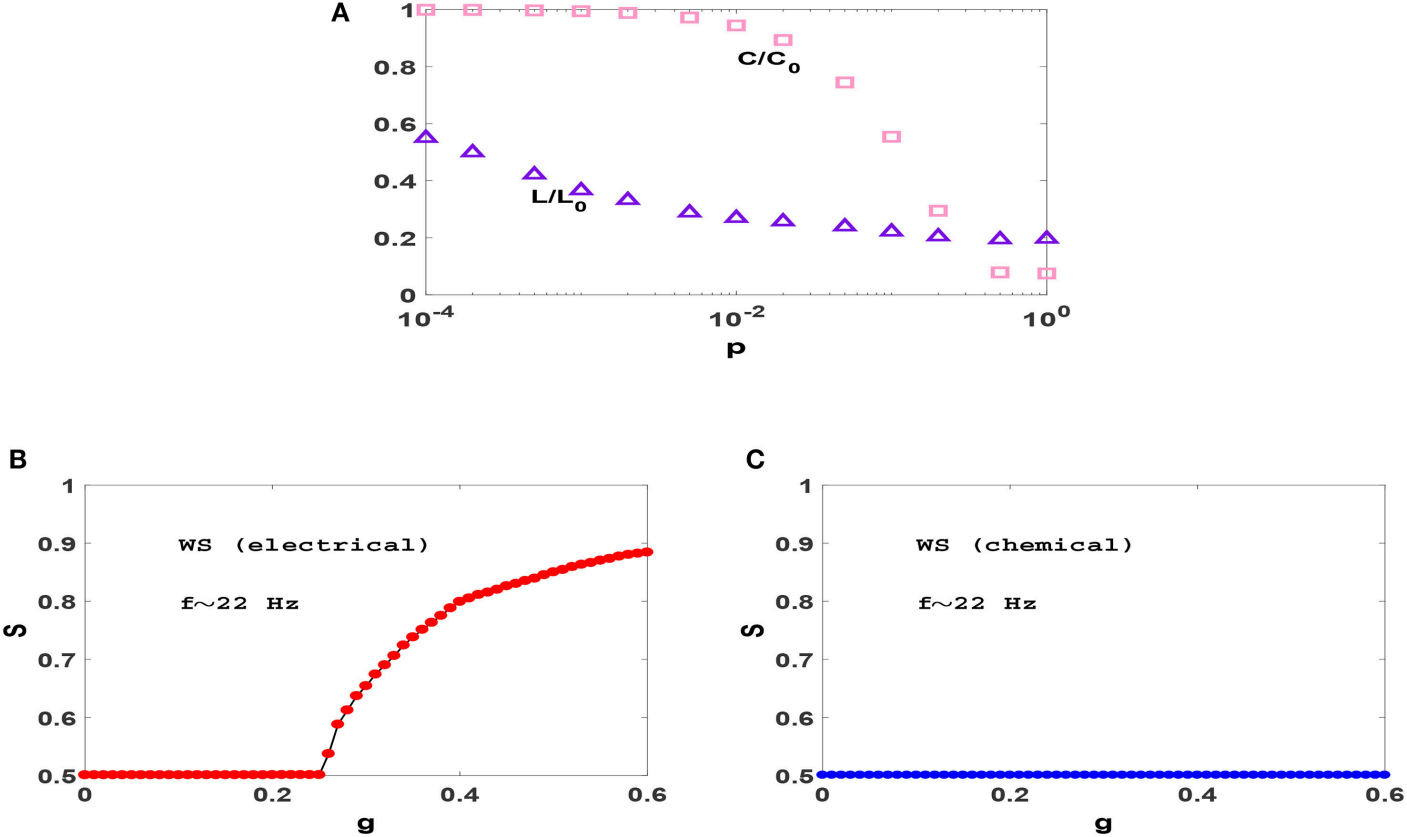
**(A)** Dependence of clustering coefficient *C* and average path length *L* on rewiring probability *p* in WS networks with *N* = 1, 000 and *z* = 50. *C* and *L* are normalized with *C*_0_ and *L*_0_ which are the clustering coefficient and average path length of a regular ring (*p* = 0), respectively. **(B,C)** Synchronization diagram of Izhikevich neurons on WS networks with *p* = 0.01, for electrical and chemical synaptic interactions. The mean firing rate is *f* ≃ 22 Hz here.

To examine this we consider transition to phase synchronization in Watts-Strogatz (WS) small-world networks (Watts and Strogatz, 1998). In Figure 3A, we have plotted the variation of clustering coefficient *C* and average distance *L* when we rewire the previous ring with different probabilities *p* and found that for *p* = 0.01 the resulting network has significant small-world effect and clustering coefficient, simultaneously. Figures 3B,C show the synchronization diagrams of Izhikevich neurons with electrical and chemical synapses in WS networks with *p* = 0.01. It is seen that the resultant synchronization diagrams are similar to *S*−*g* plots of regular ring except for a different (larger) transition point in circuits with electrical synapses. Investigation of the raster plots of WS neural networks (not shown) reveals that the underlying reason for the observed synchronization transitions is exactly the same as the reason explained for regular ring above. Since our regular ring and WS network have approximately the same value of *C* but distinctly different values of *L*, similarity of the transitions which they produce indicates that the clustering coefficient (and not the small-world effect) is the main topological factor that plays an important role in the resulting transitions.

**Figure 4:**
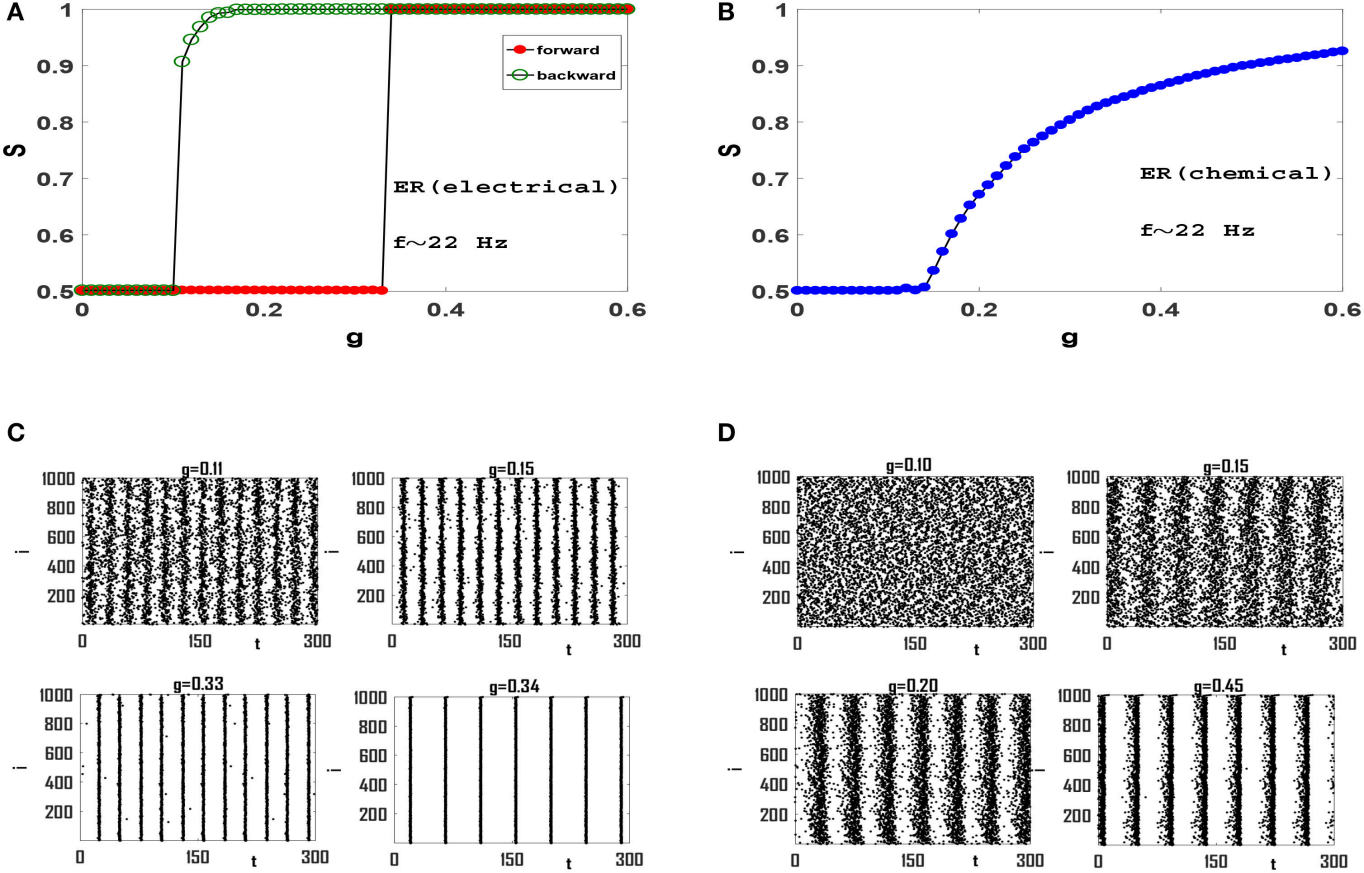
Synchronization diagram of Izhikevich neurons on ER network: **(A)** *S*−*g* plot in forward and backward evolution of the system with electrical synapses. **(B)** *S*−*g* plot for the system with chemical synapses. **(C)** Raster plots for the system with electrical synapses in forward direction. **(D)** Raster plots of the system with chemical synapses. The mean firing rate is *f* ≃ 22 Hz and *t* = 0 indicates the beginning of stationary state. *t* is measured in units of *ms*.

In order to examine the role of clustering coefficient further, we investigate more random topologies, viz, Erdos-Renyi (ER) network (Erdos and Renyi, 1959) and scale-free (SF) network with small average path length and negligible clustering coefficient (Gros, 2015). Both these networks have random structures with ER being homogeneous and SF being heterogenous exhibiting hubs which are thought to be to play an important role in synchronization phenomena (Gomez-Gardenes et al., 2011). Also, both these networks exhibit small-world effect, while the existence of hubs in SF networks leads to a relatively smaller *L* for a fixed *z* and *N*. Dependence of order parameter *S* on coupling strength *g* for an ER network of Izhikevich neurons with electrical synapses is illustrated in Figure 4A. The network exhibits a first-order or explosive transition to phase synchronization, with a large hysteresis loop, as neurons spike with beta rhythms. Note that the transition is truly explosive as *S* jumps directly to its maximum value immediately at the transition point, indication full synchrony in the network. Explosive synchronization is a novel phenomenon and has attracted much attention recently. Different mechanisms have been reported for generation of such type of synchronization transition so far (Gomez-Gardenes et al., 2011; Leyva et al., 2012; Ji et al., 2013; Skardal and Arenas, 2014; Zhang et al., 2015), and the key role played by heterogeneity has been in focus in this regard.

Seeking the underlying reason of this explosive transition, we investigate raster plots of this neural circuit for different values of *g*. Four such raster plots for forward evolution of the system are shown in Figure 4C. We find that neurons spike out-of-order initially. As *g* is increased slightly, the neurons in the system are organized into two distinct groups in which members of each group spike almost coherently, as they oscillate anti-phase with the other group. Further increase of *g* regulates neuronal phases in each group but the phase lag between two groups remain robust. Therefore there exists no global phase coherence in the system and *S* = 0.5, see *g* = 0.33 in Figure 4C. There exists a transition point for which these two anti-phase groups abruptly join together leading to complete phase coherence. Hence the order parameter suddenly jumps from *S* = 0.5 to *S* = 1, see Figure 4A and *g* = 0.34 in Figure 4C.

When interaction among neurons is mediated via the softer chemical synapses (Figure 4B), anti-phases groups do not form in the neural network. Since the clustering coefficient of ER network is negligible (*C* = 0.054 here) and long-range interaction is significant, wave-like patterns in neuron spikes do not appear. See raster plots in Figure 4D. The gradual increase of *g* subsequently leads to gradual increase in global order in the system leading to a continuous transition at which global order appear in the system, see Figure 4D. Further increase of *g* leads to increase of *S* as more and more neurons align their phases. Therefore Izhikevich neurons with mean firing rate *f* ≃ 22 Hz produce continuous transition to phase synchronization when they interact via chemical synapses on an ER network.

**Figure 5:**
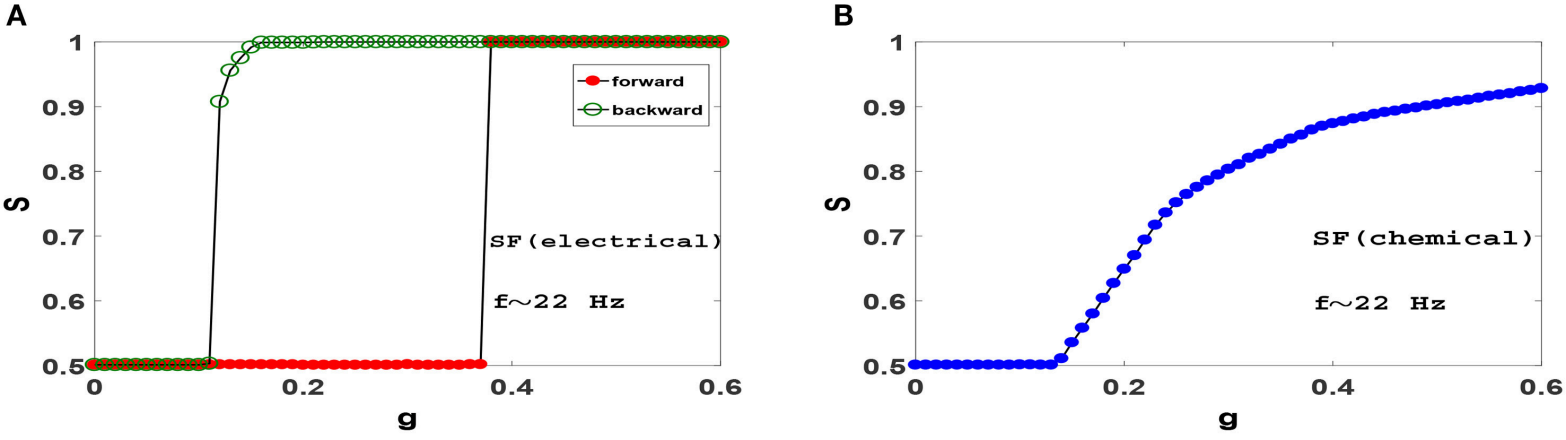
Synchronization diagram of Izhikevich neurons on SF networks with *N* = 1, 000, *z* = 20 and degree distribution function *P*(*k*)~*k*^−γ^ with γ = 3. **(A)** Phase transition diagram for the system with electrical synapses in forward and backward evolution of the system. **(B)** Phase transition diagram for the system with chemical synapses. The mean firing rate is *f* ≃ 22 Hz.

Next, we ask whether heterogeneity in SF networks can change the picture obtained from ER networks above. Figure 5 displays synchronization diagrams of Izhikevich neurons on SF networks. Here we have generated uncorrelated SF networks (Catanzaro et al., 2005) with coordination number *z* = 20 and degree distribution function *P*(*k*)~*k*^−γ^ with γ = 3. Smaller *z* is necessary here in order to give real meaning to heterogeneity needed in our study for SF networks. Note, that despite using smaller *z* the network still displays significant small-world effect and small clustering coefficient, see Table 1. *S*−*g* plots of Izhikevich neurons with mean firing rate *f* ≃ 22 Hz on SF networks with electrical and chemical synapses are illustrated in Figures 5A,B, respectively. Interestingly, it is observed that the resulting synchronization diagrams are essentially exactly the same as the results obtained for ER network. We therefore conclude that the existence of hubs does not have a significant effect in the synchronization pattern in the parameter regime we have focused for Izhikevich neurons. Furthermore, the dramatic change in clustering coefficient of random networks (ER or SF) lead to decidedly different synchronization pattern when compared to clustered networks such as regular ring or WS network.

**Figure 6:**
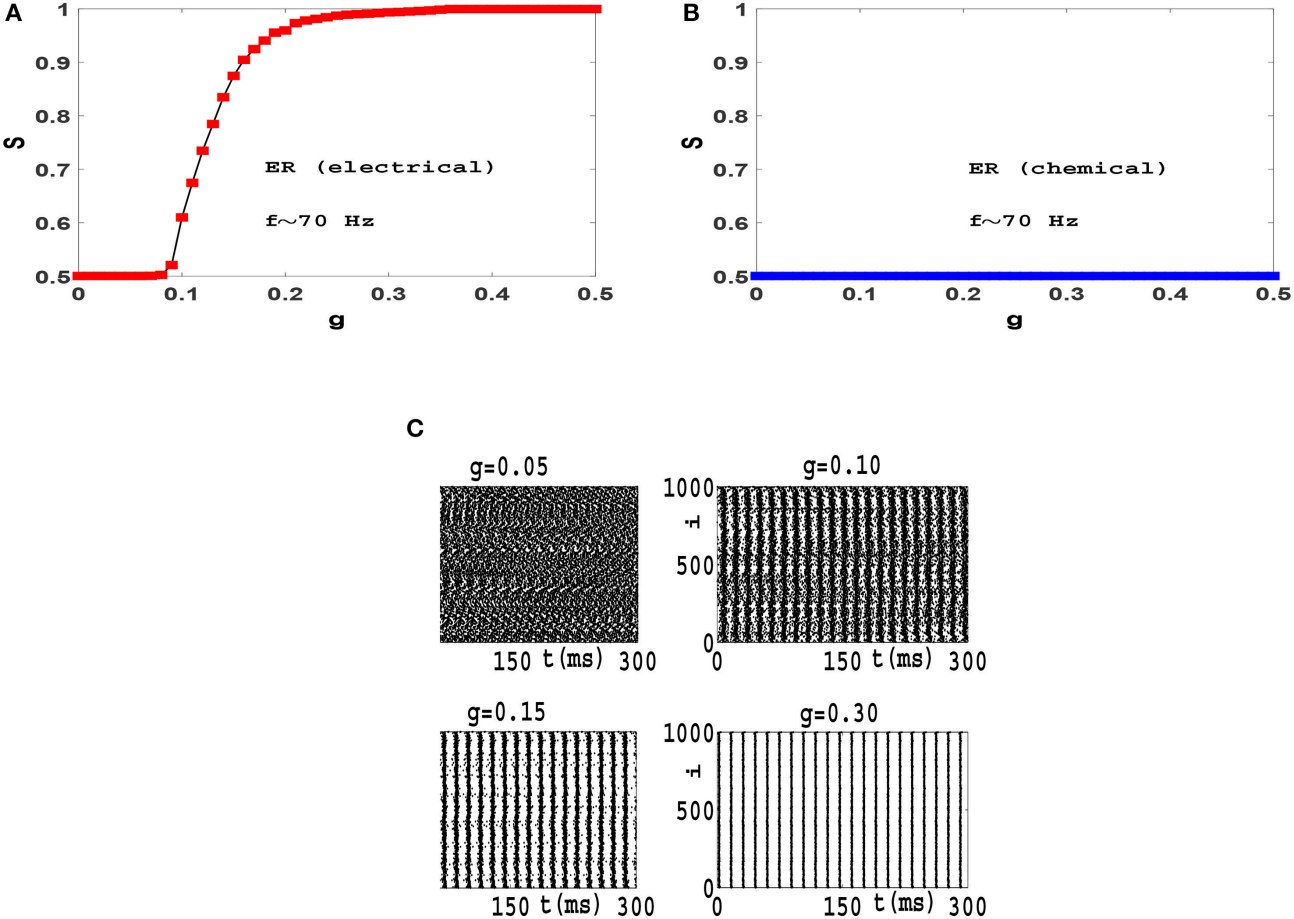
Synchronization diagram of Izhikevich neurons with spiking frequencies in the high gamma band on ER networks: Phase transition diagram for the system with **(A)** electrical synapses and **(B)** chemical synapses. **(C)** Raster plots for the system with electrical synapses. The mean firing rate is *f* ≃ 70 Hz and *t* = 0 indicates the beginning of stationary state.

When synchronization transition is studied in a population of phase oscillators such as Kuramoto model, results are independent of the mean value of frequency distribution. Therefore we can switch to a rotating frame of reference where the mean value of frequencies is zero (Gros, 2015). In contrast we found that the resulting synchronization transitions which we obtain in neural circuits depend on the mean frequency of firing. For example, in Figure 6 we have illustrated synchronization diagrams and raster plots of Izhikevich neurons with firing frequencies in high gamma band (*f* = 〈*f*_*i*_〉 ≃ 70 Hz) on ER network. Comparing these results with plots of Figure 4, it is observed that while neurons oscillate with high gamma rhythms, electrical synapses lead to a continuous transition to phase synchronization (rather than the explosive transition in beta frequencies) and chemical synapses do not lead to any synchronization in the system (as opposed to a continuous transition in beta frequencies). Also investigation of raster plots shows that while neurons fire with high gamma frequencies, interactions via electrical synapses do not results in anti-phase synchronization. (Compare Figure 4C and Figure 6C). This is a curious result that needs further investigation.

**Figure 7:**
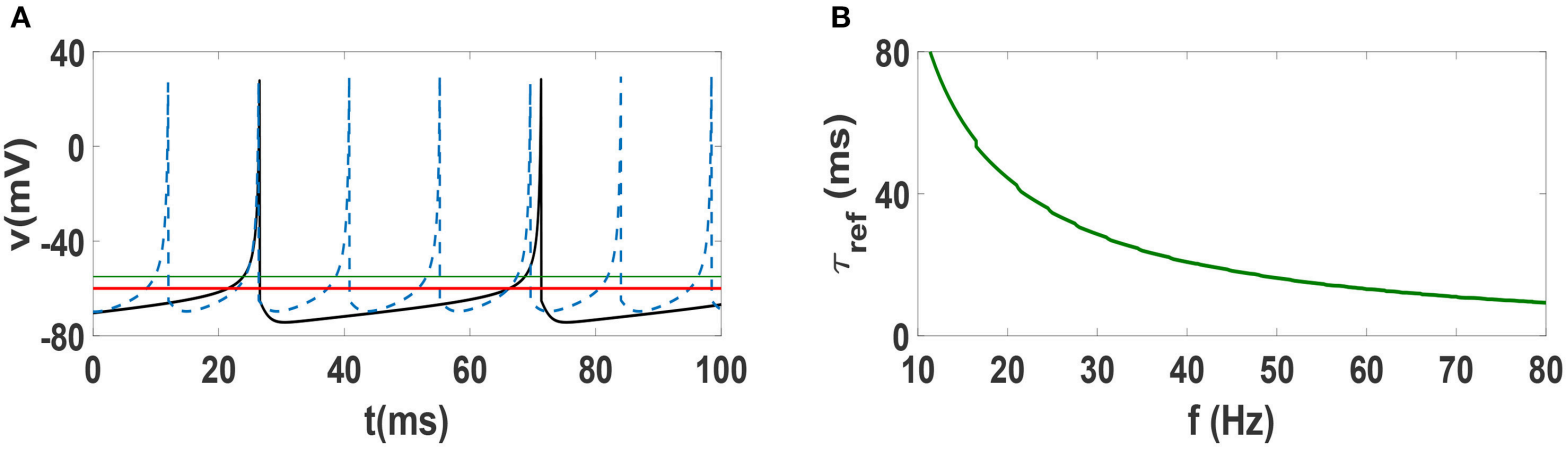
**(A)** Spike train of an Izhikevich neuron with spiking frequency 22 Hz (solid black curve) and with frequency 70 Hz (dashed blue curve). The solid green line indicate the threshold of firing and solid red line is the rest potential. **(B)** Dependence of refractory period τ_*ref*_ of Izhikevich neuron on spiking frequency. *t* = 0 indicates the beginning of stationary state.

In order to justify the frequency-dependent behavior of our neural networks, we illustrate spike trains of an individual Izhikevich neuron for two different values of firing frequencies *f* = 22 Hz and *f* = 70 Hz in Figure 7A. The horizontal solid green line at −55 mV indicates the threshold for firing, while the solid red line at −60 mV indicates the resting potential. One can see that the firing pattern of the two neurons are exactly the same, i.e., the dynamics above threshold are identical. However, the dynamics below the resting potential is decidedly different, as the lower frequency beta oscillation takes much longer to reach resting potential. Note that the hyperpolarization is stronger in the beta regime and the relative refractory period (time during which the system remains below resting potential) is clearly longer. This time scale τ_*ref*_ which renders the neuron to be relatively unexcitable is an important factor. In other words, while changing the frequency of Izhikevich neurons does not change the time scale of firing, it has a strong influence on the refractory period. This relative change of the time scales (firing vs. refractory) can provide an explanation for why anti-phase oscillations (and consequently explosive synchronization) occur in low frequency regime but not in the high frequency regime. In fact, existence of anti-phase oscillations have been attributed to separation of time scales in models of epidemic spreading (He and Stone, 2003). In Figure 7B, we plot the refractory period of Izhikevich neurons as function of frequency in the beta and gamma regime. One sees that in the Izhikevich neuron, the refractory period can become considerably long as one lowers the frequency of oscillations. While such a behavior may be an artifact of the model, one can see that many other neuronal dynamics models also exhibit similar behavior, i.e., a long time associated with slow increase in potential at low frequencies. Therefore, one might suspect that anti-phase oscillations and explosive synchronization might be associated with other generic neuronal models as well.

## 4. Discussion

Synchronization transition in a network of oscillators has attracted much attention in recent years. The Kuramoto model has been used extensively in this regard with important implications for neural networks. However, it is a very crude approximation to consider neurons as phase oscillators. In this work we have studied synchronization transition in network models of biologically plausible neurons. We used Izhikevich neurons in beta frequency range coupled with electrical and chemical synapses on various network structures. We found that stronger electrical synapses are more conducive to synchronize than chemical synapses, regardless of network structure. We also found that electrical synapses can lead to anti-phase synchronization while no such behavior was seen for chemical synapses. As far as network structure was concerned, we found that the clustering coefficient, and not the small-world effect, is the key topological factor that determines the order of synchronization transition. When we introduced short-cuts into the regular ring, no significant change in the pattern of transition was observed. However, when random networks with small clustering coefficient were considered, synchronization patterns were significantly different. Additionally, heterogeneity in network structure did not play an important role as ER and SF results were identical. We note that anti-phase synchronization leading to explosive synchronization transition is a new and interesting mechanism which has not been reported in the existing literature to the best of our knowledge. The standard mechanisms reported in the literature for explosive synchronization are associated with heterogeneity and disorder. The reported results in this work becomes more interesting when we note that explosive synchronization occurred in the beta band and was not observed in the gamma band, i.e., it is frequency-dependent and therefore of dynamical origin, as opposed to the more widely-studied structural underpinnings. We note the fact that beta and gamma rhythms have different synchronization patterns has been reported before (Kopell et al., 2000). Such a frequency dependent behavior in synchronization patterns seem important and deserves further investigation (Khoshkhou and Montakhab, submitted).

## Author contributions

AM and MK conceived the project. MK carried out the computations. AM and MK analyzed the data. AM and MK wrote the paper.

### Conflict of interest statement

The authors declare that the research was conducted in the absence of any commercial or financial relationships that could be construed as a potential conflict of interest.
